# Early-Life Stress Induces Depression-Like Behavior and Synaptic-Plasticity Changes in a Maternal Separation Rat Model: Gender Difference and Metabolomics Study

**DOI:** 10.3389/fphar.2020.00102

**Published:** 2020-02-26

**Authors:** Yongfei Cui, Kerun Cao, Huiyuan Lin, Sainan Cui, Chongkun Shen, Wenhao Wen, Haixin Mo, Zhaoyang Dong, Shasha Bai, Lei Yang, Yafei Shi, Rong Zhang

**Affiliations:** ^1^ School of Pharmaceutical Sciences, Guangzhou University of Chinese Medicine, Guangzhou, China; ^2^ School of Fundamental Medical Science, Guangzhou University of Chinese Medicine, Guangzhou, China; ^3^ School of Nursing, Guangzhou University of Chinese Medicine, Guangzhou, China

**Keywords:** early-life stress, maternal separation, synaptic plasticity, depression, metabolomics

## Abstract

More than 300 million people suffer from depressive disorders globally. People under early-life stress (ELS) are reportedly vulnerable to depression in their adulthood, and synaptic plasticity can be the molecular mechanism underlying such depression. Herein, we simulated ELS by using a maternal separation (MS) model and evaluated the behavior of Sprague–Dawley (SD) rats in adulthood through behavioral examination, including sucrose preference, forced swimming, and open-field tests. The behavior tests showed that SD rats in the MS group were more susceptible to depression- and anxiety-like behaviors than did the non-MS (NMS) group. Nissl staining analysis indicated a significant reduction in the number of neurons at the prefrontal cortex and hippocampus, including the CA1, CA2, CA3, and DG regions of SD rats in the MS group. Immunohistochemistry results showed that the percentages of synaptophysin-positive area in the prefrontal cortex and hippocampus (including the CA1, CA2, CA3, and DG regions) slice of the MS group significantly decreased compared with those of the NMS group. Western blot analysis was used to assess synaptic-plasticity protein markers, including postsynaptic density 95, synaptophysin, and growth-associated binding protein 43 protein expression in the cortex and hippocampus. Results showed that the expression levels of these three proteins in the MS group were significantly lower than those in the NMS group. LC–MS/MS analysis revealed no significant differences in the peak areas of sex hormones and their metabolites, including estradiol, testosterone, androstenedione, estrone, estriol, and 5β-dihydrotestosterone. Through the application of nontargeted metabolomics to the overall analysis of differential metabolites, pathway-enrichment results showed the importance of arginine and proline metabolism; pantothenate and CoA biosyntheses; glutathione metabolism; and the phenylalanine, tyrosine, and tryptophan biosynthesis pathways. In summary, the MS model caused adult SD rats to be susceptible to depression, which may regulate synaptic plasticity through arginine and proline metabolism; pantothenate and CoA biosyntheses; glutathione metabolism; and phenylalanine, tyrosine, and tryptophan biosyntheses.

## Highlights

1. A maternal separation model was used to study the effects of early-life stress on adult depression-like behavior.
2. The model induced depression-like behavior in adult Sprague–Dawley rats, but no statistical significance difference was found in gender.
3. Maternal separation causes synaptic-plasticity changes.
4. Metabolomics studies indicated the importance of arginine and proline metabolism; pantothenate and CoA biosyntheses; glutathione metabolism; and the phenylalanine, tyrosine, and tryptophan biosynthesis pathways.

## Introduction

As a common mental disorder worldwide, depression has affected more than 300 million people of all ages globally (World Health Organization (WHO), 2019), causing heavy financial burden on families and the society. The occurrence of depression is closely related to childhood exposure to adverse stress (Saleh et al., 2017). Children under the influence of early-life stress (ELS), including childhood abuse and parental neglect, have considerably high probabilities of developing emotional and mental illnesses (Anacker et al., 2014; Menard et al., 2016), including anxiety and depression (Targum and Nemeroff, 2019; United States Centers for Disease Control and Prevention, 2019). In gender, the incidence of depression in women is as twice as that in men (Kessler et al., 2003), given that impaired neuronal functions by sex hormone fluctuations lead to depressive symptoms (Bloch et al., 2003; Duman et al., 2016). However, a meta-analysis by Salk RH et al. showed that gender differences in depression incidence peak during adolescence, whereas the gender gap narrows and stabilizes in adulthood (Salk et al., 2017). Simultaneously, ELS may increases depressive-like behavior, affect hippocampal neurogenesis, and cause mild metabolic imbalance in early adulthood (Ruiz et al., 2018). Maternal separation (MS), as an ELS event model for rodents, indicates that pups exposed to MS environment display passive–submissive behavior and passively cope with stressful behavior during adulthood (Gardner et al., 2005), have long-term disruption on neural development, and may underlie vulnerability to depression (Hanson et al., 2012; Stuart et al., 2019; Zheng et al., 2019).

The specific molecular mechanism of depression remains unclear because of its complex pathogenesis. The regulation of synaptic plasticity is closely related to the induction of depressive disorders. Neuronal atrophy, synaptic loss, and reduction of synaptic density have been investigated in studies on synaptic dysfunction in depression (Duman and Aghajanian, 2012; Duman et al., 2016). Brain structural plasticity, shrinkage of CA3 dendrites and dentate gyrus neurons, and spine loss in CA1 neurons occur in the hippocampus with induced chronic stress (McEwen, 1999). Depression rodent models show loss of spines and dendrites, weakened synapse function, and decreased quantity of synapse in the prefrontal cortex (Kang et al., 2012). The number and size of dendritic spines reflect the changes in synaptic plasticity (Colgan and Yasuda, 2014). Postsynaptic density 95 (PSD-95) is a membrane-associated guanylate kinase family scaffolding protein at the postsynapse that plays a key role in synaptic plasticity (Xu, 2011; Wu et al., 2017). Synaptophysin (SYN), which is extensively distributed in the presynaptic vesicle membrane, is a calcium-binding glycoprotein and is closely related to synaptic plasticity because its expression demonstrates synaptic density, distributed area, and functional state (Zhu et al., 2019). Growth-associated binding protein 43 (GAP-43) is a neuron-specific and membrane-associated phosphoprotein, and its expression is relevant to synaptic plasticity, neuronal development, and regeneration (Goslin et al., 1988; Zhu et al., 2019). PSD-95, SYN, and GAP-43 proteins are markers of synaptic plasticity in depressive disorder (Reines et al., 2008).

Metabolomics is extensively used to investigate depression. Metabolomics focuses on holistic analysis by evaluating endogenous metabolites with molecular masses lower than 1000 Da (Wang et al., 2015; Wang et al., 2016). For untargeted metabolomics, statistically significant differential metabolites are selected in all detected metabolites in the sample by comparing the model and control groups, revealing the comprehensive metabolism of a whole tissue (Zou et al., 2013). Depression is closely related to the imbalance of amino acid metabolism, lipid metabolism, and energy metabolism in both clinical research (Zheng et al., 2012) and animal experiments (Liu et al., 2016; Zhang et al., 2018). Gultyaeva et al. reported that neuronal structure restoration is disrupted in depression by stimulating biochemical pathways (Gultyaeva et al., 2019).

In this study, we aimed to explore the mechanism of the MS rat model in depression-like behavior in adult rats. In summary, we explored the relationship between synaptic plasticity and depression and studied the underlying mechanisms of depression by regulating synaptic plasticity on MS rat model by using metabolomic research. Simultaneously, the effect of gender differences on synaptic plasticity was studied.

## Materials and Methods

### Animals

Male and female SD rats were purchased from the Animal Experimental Center of Guangzhou University of Chinese Medicine. The rats were housed in standard polypropylene cages with food and water available *ad libitum*. Sterilized wood shavings were used for bedding. The cages were maintained in a 12/12 h reversed light/dark cycle. Lights were on at 20:30, and the cages were kept under controlled temperature (20–26 °C) and humidity (40%–70%). This study was carried out in accordance with the principles of the Basel Declaration and approved by the Committee of Animal Experiment Ethics Review in Guangzhou University of Chinese Medicine. All efforts were made to minimize the suffering of the animals.

### MS

Male and female rats were mated to produce a litter of 8–12 pups, and the day of birth was defined as postnatal day (PND) 0. All litters of each dam were divided into F-NMS, F-MS, M-NMS, and M-MS groups, which include a total of 15 pups per group. From PND1 to PND21, the pups of the MS groups were separated from their dams daily (from 08:00 to 11:00 and from 14:00 to 17:00). Each of the pups was transferred to a box filled with bedding obtained from dam's cage, placed on cotton maintained at 30°C–33°C, and returned to the cage together with their dams. At the same time, the NMS groups were left undisturbed as the control group. Pups were weaned after PND21, and male and female rats were randomly redistributed to eight pups per cage. The body weight of rats was measured and recorded once a week from PND28 to PND63. Behavioral tests were conducted from PND56 to PND63, and animals were sacrificed at PND63. Per group, 6 rats were subjected to WB, 3 rats for Nissl staining and immunohistochemistry, and 6 rats for untargeted metabolomic analysis. All of these rats were selected randomly.

### Behavioral Examination

#### Sucrose Preference Test

As a core component of depression, anhedonia is assessed through sucrose preference test. First, the rats were individually placed in cages and acclimated to two bottles of 1% sucrose solution. They were presented with 1% sucrose solution after 24 h, the drinking water was replaced, and their positions were exchanged after 12 h. Subsequently, the rats were deprived of food and water for 24 h before the test day, and free access was provided to two identical bottles, including 1% sucrose and normal drinking water from 08:00 to 10:00. Finally, the consumption of sucrose solution and drinking water and sucrose preference (%) were measured on the basis of the percentage of sucrose consumption relative to the sum of sucrose and water consumption.

#### Open-Field Test

Anxiety is assessed through open-field test. In this procedure, the rats were individually placed at the center of an open-field apparatus for 3 min. The time spent in the center, distance traveled in the center, and activity were analyzed using a computer linked to the camera above the open-field apparatus as an indicator of anxiety.

#### Forced Swimming Test

The forced swimming test is applied to assess depression behavior. On the day before the test, the rats were forced to swim for 15 min separately in cylinders under a water temperature of 21–25°C and a depth of 23 cm. In the second session, the duration time of immobility was recorded during 5 min of the test.

### Nissl Staining and Immunohistochemistry

When all of the behavioral tests were completed, rats' brain were taken out, placed on ice, and then transcardially perfused with saline followed by 4% paraformaldehyde. The whole brain was embedded in wax and cut into 5 μm-thick coronal sections. For Nissl staining, the sections were dewaxed with xylene (3 times for 30 min), graded alcohol solutions (100%, 90%, 70%, once for 5, 2, and 2 min, respectively), and distilled water (once for 5 min). Subsequently, tissue slices were stained by toluidine blue (Beyotime Biotechnology, Shanghai, China) for 1 h at a 50°C environment. The slides were rinsed with distilled water (twice for 10 s), 95% alcohol (twice for 4 min, secondary time reagent was cleaned), and xylene (twice for 10 min, secondary time reagent was cleaned). The total number of cells in the prefrontal cortex area and CA1, CA2, CA3, and DG area in the hippocampus was observed and counted using a 400× field optical microscope. ImageJ (version 1.45) was used to count the number of nerve cells.

For immunohistochemistry, the sections were incubated with 3% H_2_O_2_ for 10 min and 10% goat serum for 15 min after deparaffinization with xylene and graded alcohol (100%, 95%, 90%, 80%, 70%). The antigen was retrieved and incubated with primary antibody (SYN, 1:200, Affinity, USA) overnight at 4°C. Subsequently, secondary antibody goat-anti-mice IgG was conjugated for 15 min at room temperature. The color of the sections was developed with DAB and counterstained with hematoxylin. The percentage of positive area of SYN protein was statistically measured on ImageJ (version 1.45).

### WB Analysis

First, the regions of dissection were identified using the *Color Atlas of Comparative Histology of Laboratory Animals.* Rats were dissected on ice as follows. The skull was opened, the first incision is made at the end of the hemisphere. The second incision was made into the lateral ventricle in front of the first incision. Both incisions reached the ventral of the brain. The cerebral cortex covering the hippocampus was then taken out. After exposing the hippocampus, the other side of the brain was processed. Both sides of the cortex covering the hippocampus along the ventricle was pulled up, and the rest of the hippocampus from the cortex covering it along the surface of the hippocampus towards the ventral part of the hippocampus was separated. The hippocampus was taken off, and the hippocampus and cortex were stored individually in liquid nitrogen. The tissues were used for WB analysis.

The lysate of hippocampal and cortical tissues was centrifuged at 12,000 rpm for 20 min at 4°C. The supernatant was quantified with BCA Protein Assay Kit (KeyGEN BioTECH). Approximately 30 μg of total protein was separated with 12% SDS-polyacrylamide gel electrophoresis and transferred to polyvinylidene fluoride membranes. The membranes were blocked with 5% skim milk powder for 2 h and incubated with primary antibody overnight at 4°C. Subsequently, the membranes were incubated with the corresponding secondary antibody for 1 h at room temperature. The bound proteins were detected using a BIO-RAD imaging system (BIO-RAD, Hercules, CA, USA). The grayscale values of each band relative to tubulin from the same sample were analyzed on Image Lab (Millipore, USA). The primary antibodies for immunoblotting were as follows: PSD-95 (1:1000, Affinity, USA), GAP-43 (1:1000, Affinity, USA), SYN (1:1000, Affinity, USA), and tubulin (1:5000, Affinity, USA).

The three synaptic-plasticity proteins SYN, PSD-95, and GAP-43 detected in this study have similar molecular weights and cannot be detected simultaneously. For the small amount of hippocampal tissue, the hippocampal tissue of each rat is insufficient to complete the detection of all proteins. Therefore, we randomly selected three tissues for SYN, PSD95, and tubulin detection, and the 3 remaining tissues were used for GAP43 and tubulin detection. The corresponding cortex of the rats was selected for the detection of the same protein to maintain the parallelism of the experiment.

### Untargeted Metabolomics Analysis

#### Brain Tissue Sample Preparation

Approximately 500 μL of 70% precooled methanol was added into 50 mg of brain tissue. Centrifugation (12 000 rpm, 10 min) was performed at 4°C after vortex and sonication to obtain the supernatants. Furthermore, 500 μL of ethyl acetate/methanol (v, 1:3) was added into the precipitate, and the previous steps were repeated. Two aliquots of the above supernatants were mixed and concentrated. Subsequently, 100 μl of 70% methanol was added to the powder, sonicated for 3 min, and centrifuged (12 000 rpm, 3 min) at 4°C.

#### UPLC-QTOF-MS Analysis

Approximately 60 μL of sample supernatant was injected into Waters T3 C18 (2.1 mm × 100 mm, 1.8 μm) and maintained at 25°C by using an Agilent 1290 Infinity LC UPLC system coupled to an Aglient-QTOF/MS-6545 mass spectrometer. Mobile phase A comprised 0.01% formic acid/water. Mobile phase B was composed of acetonitrile.

#### Data Preprocessing and Multivariate Analysis

The raw data files obtained by LC–MS/MS analysis were first extracted on the Profinder software (Agilent) to obtain information, such as mass-to-charge ratio, retention time, and peak area of the characteristic peaks. Pareto-scaled data were imported into the Metaboanalyst (http://www.metaboanalyst.ca) and SIMCA-P software (version 14.1. Umetrics, Umea, Sweden). Partial least squares discriminant analysis (PLS-DA) and orthogonal PLS-DA analysis (OPLS-DA) were performed in the NMS and MS groups. The OPLS-DA model was validated through 200-iteration permutation tests. The criterion for identifying significant differential metabolites was variable importance in the projection (VIP) > 1 and *p* < 0.05 in the OPLS-DA model. Selected differential metabolites were used for pathway enrichment, and the selection criteria were FDR < 0.05 and impact > 0.

### Statistical Analysis

All experimental data were presented as mean ± standard error of the mean. The main effects of two levels of treatment (NMS or MS), two levels of sex (male and female), and treatment × sex interaction were analyzed by two-way ANOVA. Student's t-test was performed when main effects were found. Differences were considered statistically significant at *P* < 0.05. Statistical analyses were performed on SPSS version 22.0 (Chicago, IL, USA) and GraphPad Prism (La Jolla, CA, USA).

## Results

### MS Reduced the Body Weight of SD Rats

**Figure 1:**
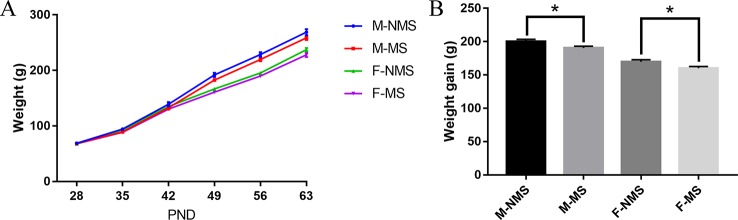
MS reduces the body weight of SD rats. **(A)** The tendency of weight gain from PND28 to PND63. **(B)** Weight gain from PND28 to PND63 after MS. Statistical analyses are performed by two-way ANOVA followed by t-test. Values are presented as mean ± SEM, *p < 0.05. *n* = 15 per group.

As shown in **Figure 1A**, the trend of body weight change from PND28 to PND63 was observed. For weight gain from PND28 to PND63, significant main effects of treatment (*F*_(1,56)_ = 10.567, *p* = 0.002) and sex (*F*_(1,56)_ = 105.623, *p* < 0.001) were found, but no significant treatment × sex interaction were detected (*F*_(1,56)_ < 0.001, *p* = 0.988). Student's t-test showed significant reduction in the M-MS and F-MS groups relative to the M-NMS (*t*_(28)_ = 2.245, *p* = 0.033) and F-NMS groups (*t*_(28)_ = 2.357, *p* = 0.026. **Figure 1B**).

### MS Caused Depression-Like and Anxiety-Like Behaviors in SD Rats

**Figure 2:**
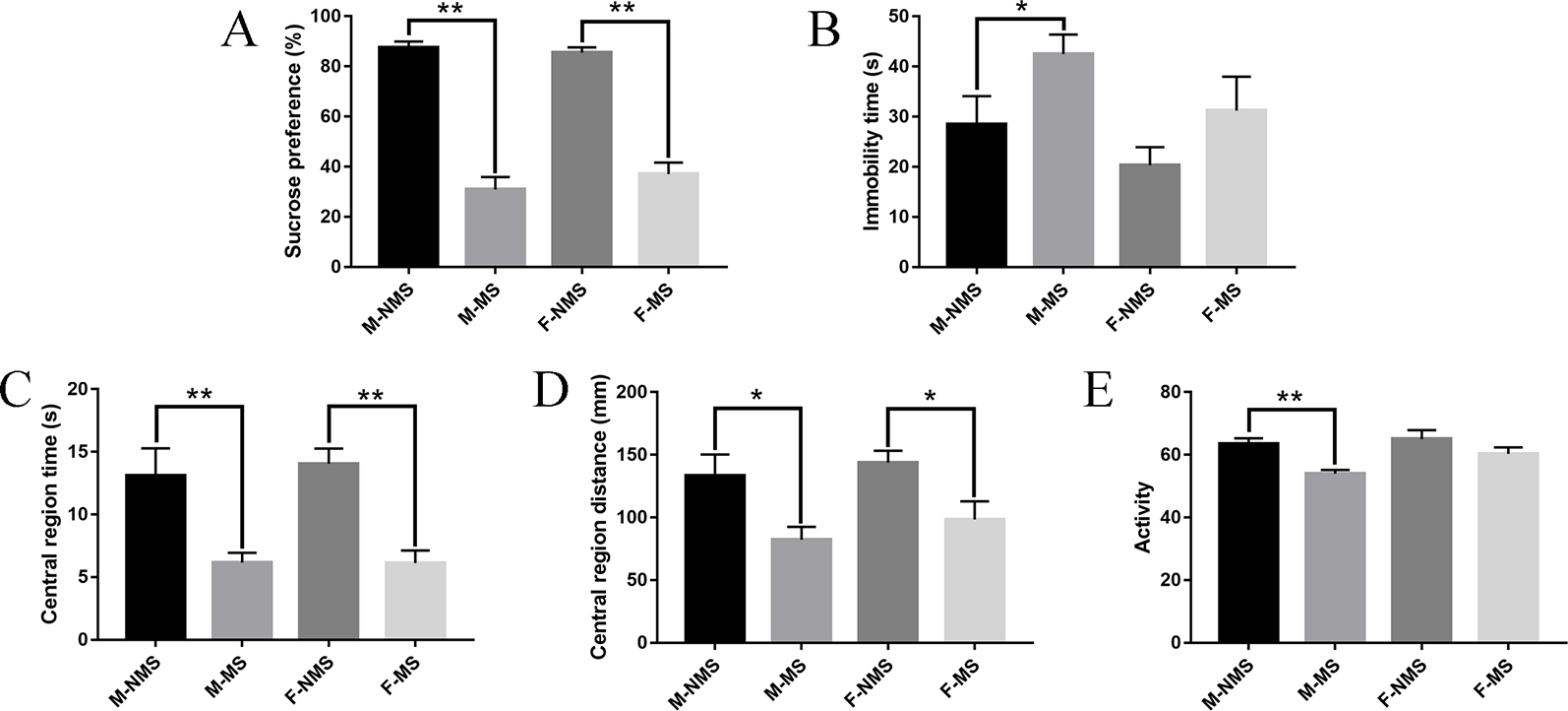
MS causes depression-like and anxiety-like behavior in SD rats. **(A)** Effect of MS on sucrose preference (%) in the sucrose-preference test on SD rats. **(B)** Effect of MS on immobility time(s) in the forced-swimming test on rats. **(C–E)** Effect of MS on central region time (s), central region distance (mm), and activity in the open-field test on rats. Statistical analyses are performed by two-way ANOVA followed by t-test. Values are presented as mean ± SEM. *p < 0.05, **p < 0.01 (compared with the NMS group), *n* = 15 per group.

In the behavioral test results, the sucrose preference test was performed to assess the anhedonia and depressive-like behavior of rats. For the sucrose preference, we did not find significant differences in sex effects (*F*_(1,56)_ = 0.339, *p* = 0.563) and treatment × sex interaction (*F*_(1,56)_= 1.170, *p* = 0.284). Significant difference was found in the main effects of treatment (*F*_(1,56)_ = 196.530, *p* < 0.001). Student's t-test results indicated that the sucrose preference in M-MS and F-MS group significantly decreased relative to that in the M-NMS (*t*_(28)_ = 10.119, *p* < 0.001) and F-NMS (*t*_(28)_ = 9.716, *p* < 0.001. **Figure 2A**) groups. The immobility time of forced swimming was used to evaluate the behavioral despair of rats, and a significant treatment effect (*F*_(1,56)_ = 5.038, *p* = 0.018) was demonstrated. No significant sex effect (*F*_(1,56)_ = 3.622, *p* = 0.062) and treatment × sex interaction (*F*_(1,56)_ = 0.091, *p* = 0.764) was detected. Student's t-test results showed that the immobility time in M-MS group significantly increased compared with the M-NMS group (*t*_(28)_ = 2.062, *p* = 0.049). However, no statistical significance was found between the F-NMS and F-MS groups (*t*_(28)_ = 1.428, *p* = 0.164. **Figure 2B**). For the assessment of anxiety-like behavior of rats with open-field test, no significant sex effect (*F*_(1,56)_ = 0.105, *p* = 0.747; *F*_(1,56)_ = 1.014, *p* = 0.318; *F*_(1,56)_ = 3.651, *p* = 0.061, respectively) and treatment × sex interaction (*F*_(1,56)_ = 0.121, *p* = 0.729; *F*_(1,56)_ = 0.048, *p* = 0.827; *F*_(1,56)_ = 1.358, *p* = 0.249, respectively) were detected in the central region time, central region distance, and activity. Treatment effect showed significant differences (*F*_(1,56)_ = 28.545, *p* < 0.001; *F*_(1,56)_ = 13.320, *p* = 0.001; *F*_(1,56)_ = 12.089, *p* = 0.001, respectively). Student's t-test results showed that the central region time and distance both in M-MS and F-MS groups were observed significantly lower relative to those of the M-NMS and F-NMS groups (*t*_(28)_ = 3.021, *p* = 0.005 and *t*_(28)_ = 5.056, *p* < 0.001 for central region time; *t*_(28)_ = 2.570, *p* = 0.016 and *t*_(28)_ = 2.605, *p* = 0.016 for central region distance; **Figures 2C, D**). The activity in the M-MS group significantly decreased compared with that in the M-NMS group (*t*_(28)_ = 4.546, *p* < 0.001). However, no statistical significance was found between the F-NMS and F-MS groups (*t*_(28)_ = 1.344, *p* = 0.191; **Figure 2E**).

### MS Decreased the Number of Neurons

**Figure 3:**
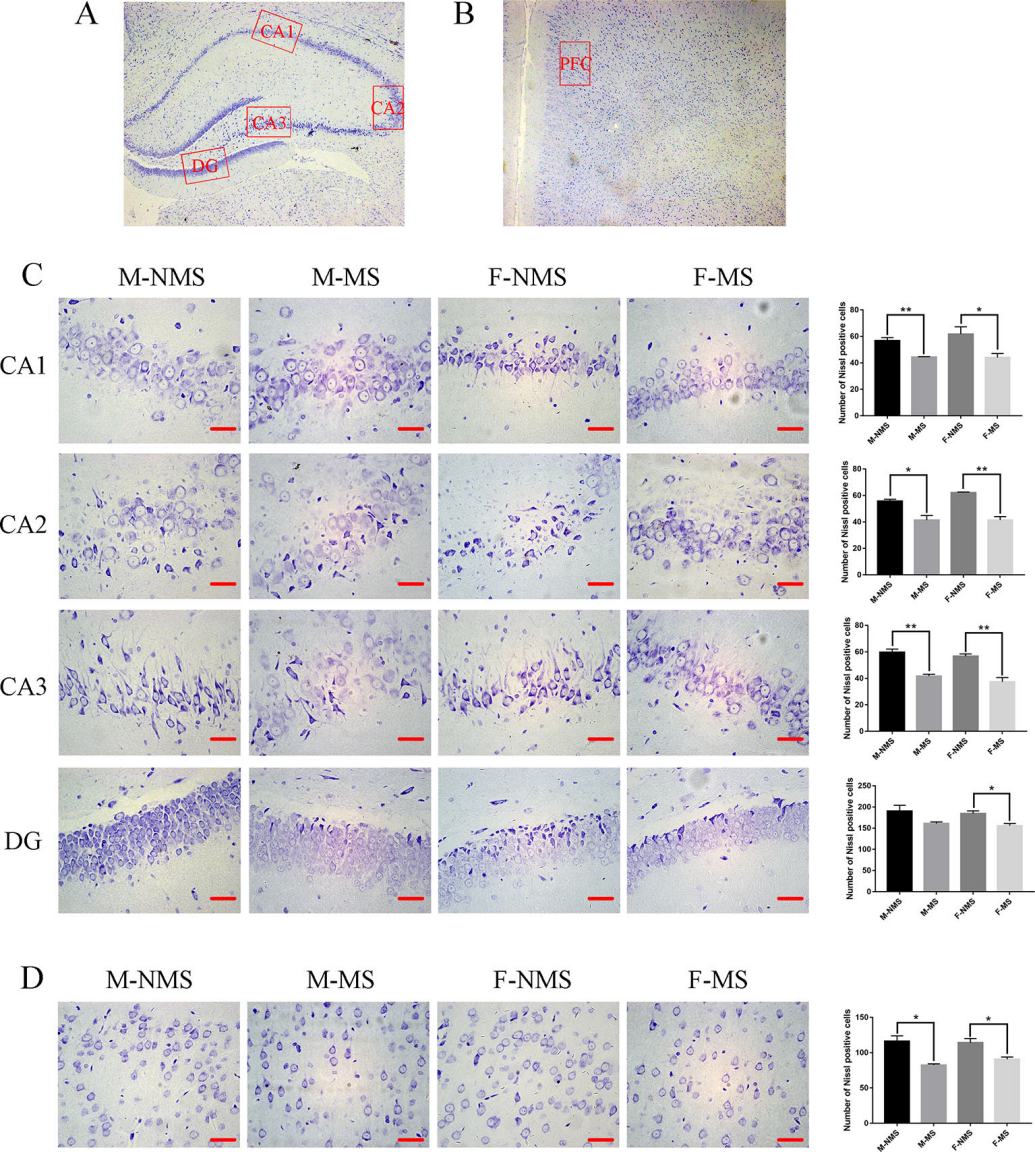
MS decreases the number of neurons. **(A)** Schematic of the coronal section from rat hippocampus and the locations of CA1, CA2, CA3, and DG regions. **(B)** The red frame area indicates the field of view of the prefrontal cortex. **(C)** Representative 400× photomicrographs of Nissl staining in the hippocampal of CA1, CA2, CA3, and DG regions. Results of the number of Nissl staining positive cells in the hippocampal CA1, CA2, CA3, and DG regions are statistically significant, except for the decreasing trend of the M-MS group in the DG region. **(D)** Representative 400× photomicrographs of Nissl staining in the prefrontal cortex. Statistical results of the number of Nissl-positive cells in the prefrontal cortex. Statistical analyses are performed by two-way ANOVA followed by t-test. Data are presented as mean ± SEM, *p < 0.05, **p < 0.01, *n* = 3 per group, scale bar = 50 µm.

Nissl staining demonstrated that the neurons were more loosely arranged and hypochromic in the M-MS and F-MS groups compared with M-NMS and F-NMS groups, as shown in **Figures 3A**–**D**. The number of neurons in CA1, CA2, CA3, and DG area of hippocampus and prefrontal cortex showed no significant difference in sex effect (*F*_(1,8)_ = 0.480, *p* = 0.508; *F*_(1,8)_ = 1.823, *p* = 0.214; *F*_(1,8)_ = 2.469, *p* = 0.155; *F*_(1,8)_ = 0.432, *p* = 0.530; *F*_(1,8)_ = 0.364, *p* = 0.563) and treatment × sex interaction (*F*_(1,8)_= 0.627, *p* = 0.451; *F*_(1,8)_ = 1.823, *p* = 0.214; *F*_(1,8)_ = 0.082, *p* = 0.782; *F*_(1,8)_ < 0.001, *p* = 0.985; *F*_(1,8)_ = 0.969, *p* = 0.354). However, significant difference was found in treatment effect (*F*_(1,8)_ = 19.853, *p* = 0.002; *F*_(1,8)_ = 55.682, *p* < 0.001; *F*_(1,8)_ = 64.000, *p* < 0.001; *F*_(1,8)_ = 11.867, *p* = 0.009; *F*_(1,8)_ = 29.477, *p* = 0.001). Student's t-test results showed a significant reduction of neurons in the CA1, CA2, and CA3 areas of hippocampus and prefrontal cortex in the M-MS group relative to the M-NMS group (*t*_(4)_ = 5.233, *p* = 0.006; *t*_(4)_ = 3.801, *p* = 0.019; *t*_(4)_ = 6.409, *p* = 0.003; *t*_(4)_ = 4.216, *p* = 0.014). However, no significant difference was found in the DG area of M-MS rats relative to the M-NMS group (*t*_(4)_ = 2.006, *p* = 0.168). In addition, neuron amount in the CA1, CA2, CA3, and DG area of hippocampus and prefrontal cortex in F-MS group was significantly lower than that in the F-NMS group (*t*_(4)_ = 2.801, *p* = 0.049; *t*_(4)_ = 7.410, *p* = 0.002; *t*_(4)_ = 5.188, *p* = 0.007; *t*_(4)_ = 3.397, *p* = 0.027; *t*_(4)_ = 3.424, *p* = 0.027; **Figures 3C, D**).

### MS Reduced SYN Protein Expression in the Hippocampus and Prefrontal Cortex

**Figure 4:**
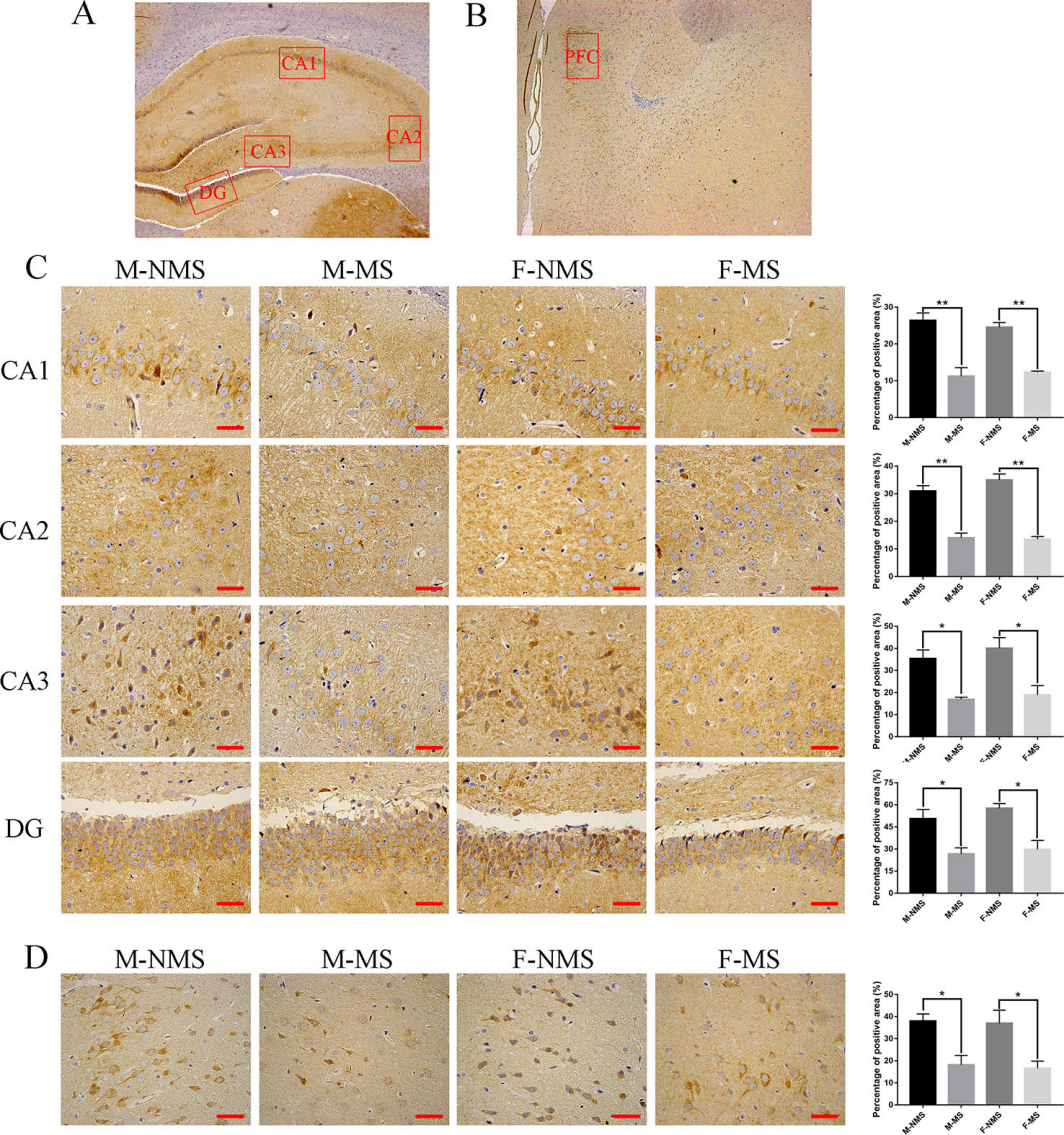
MS reduces SYN protein expression in immunohistochemistry. **(A)** Schematic of the coronal section from rat hippocampus and the locations of CA1, CA2, CA3, and DG regions. **(B)** The red frame area indicates the field of view of the prefrontal cortex. **(C)** Representative 400× photomicrographs of SYN protein expression of the CA1, CA2, CA3, and DG regions of the hippocampus. Results of the percentage of SYN-positive area in the hippocampal CA1, CA2, CA3, and DG regions are statistically significant. **(D)** Representative 400× photomicrographs of SYN protein expression in the prefrontal cortex. Statistical results of the percentage of SYN-positive area in the prefrontal cortex. Statistical analyses are performed by two-way ANOVA followed by t-test. Data are presented as mean ± SEM, *p < 0.05, **p < 0.01, *n* = 3 per group, scale bar = 50 µm.

We evaluated the expression of SYN in the CA1, CA2, CA3, and DG areas of the hippocampus and prefrontal cortical area with immunohistochemistry (**Figures 4A**–**D**). The percentage of positive area showed that the SYN expression did not display significant difference in sex effect (*F*_(1,8)_ = 0.059, *p* = 0.814; *F*_(1,8)_ = 0.996, *p* = 0.348; *F*_(1,8)_ = 0.760, *p* = 0.409; *F*_(1,8)_ = 1.017, *p* = 0.343; *F*_(1,8)_ = 0.084, *p* = 0.779) and treatment × sex interaction (*F*_(1,8)_= 0.751, *p* = 0.411; *F*_(1,8)_ = 1.716, *p* = 0.227; *F*_(1,8)_ = 0.127, *p* = 0.731; *F*_(1,8)_ = 0.158, *p* = 0.701; *F*_(1,8)_ = 0.004, *p* = 0.953). However, significant difference was found in treatment effect (*F*_(1,8)_ = 67.120, *p* < 0.001; *F*_(1,8)_ = 120.317, *p* < 0.001; *F*_(1,8)_ = 27.549, *p* = 0.001; *F*_(1,8)_ = 26.481, *p* = 0.001; *F*_(1,8)_ = 21.893, *p* = 0.002). Student's t-test revealed an obviously decreased SYN level in M-MS group compared with M-NMS (*t*_(4)_ = 4.924, *p* = 0.008; *t*_(4)_ = 6.588, *p* = 0.003; *t*_(4)_ = 4.641, *p* = 0.034; *t*_(4)_ = 3.221, *p* = 0.032; *t*_(4)_ = 3.708, *p* = 0.021), as well as in F-MS group (*t*_(4)_ = 9.338, *p* = 0.001; *t*_(4)_ = 9.027, *p* = 0.001; *t*_(4)_ = 3.298, *p* = 0.030; *t*_(4)_ = 4.102, *p* = 0.015; *t*_(4)_ = 3.029, *p* = 0.039).

### MS reduced the Expression of Synaptic-Plasticity Protein

**Figure 5:**
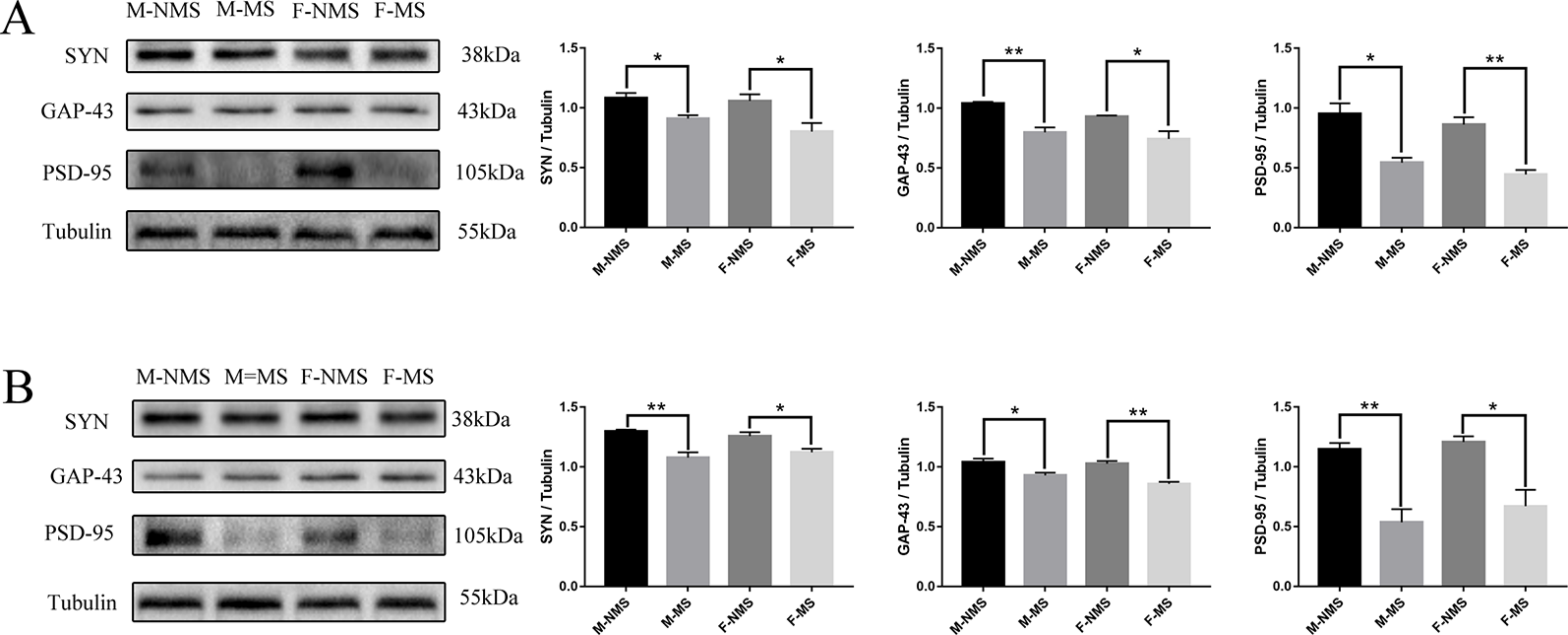
MS reduces the expression of synaptic-plasticity protein. **(A)** The bands of synaptic-plasticity proteins of SYN, PSD-95, and GAP-43 in the hippocampus by WB. Statistical results indicate the relative protein levels expressed by SYN, GAP-43, and PSD-95. **(B)** The bands of synaptic-plasticity proteins of SYN, PSD-95, and GAP-43 in cortex by WB. Statistical results indicate the relative protein levels expressed by SYN, GAP-43, and PSD-95. Statistical analyses are performed by two-way ANOVA followed by t-test. Data are presented as mean ± SEM, *p < 0.05, **p < 0.01, *n* = 3 per group.

In the hippocampus, western blot results showed that the expression of SYN (sex effect: *F*_(1,8)_ = 1.630, *p* = 0.238; treatment effect: *F*_(1,8)_ = 16.906, *p* = 0.003; treatment × sex interaction: *F*_(1,8)_ = 0.669, *p* = 0.437), GAP-43 (sex effect: *F*_(1,8)_ = 4,341, *p* = 0.071; treatment effect: *F*_(1,8)_ = 29.880, *p* = 0.001; treatment × sex interaction: *F*_(1,8)_ = 0.472, *p* = 0.512), and PSD-95 (sex effect: *F*_(1,8)_ = 2.572, *p* = 0.147; treatment effect: *F*_(1,8)_ = 48.240, *p* < 0.001; treatment × sex interaction: *F*_(1,8)_ = 0.003, *p* = 0.955) significantly differed in treatment effect and not statistically different in sex effect and treatment × sex interaction. Moreover, the expression of SYN (male: *t*_(4)_ = 3.367, *p* = 0.028; female: *t*_(4)_ = 2.826, *p* = 0.048; **Figure 5A**), GAP-43 (male: *t*_(4)_ = 5.602, *p* = 0.005; female: *t*_(4)_ = 2.860, *p* = 0.046; **Figure 5A**), and PSD-95 (male: *t*_(4)_ = 4.304, p = 0.013; female: *t*_(4)_ = 5.837, p = 0.004; **Figure 5A**) was significantly reduced in the M-MS and F-MS groups relative to the M-NMS and F-NMS groups. The protein marker levels of synaptic plasticity in the hippocampus were similar to that in the cortex: SYN (sex effect: *F*_(1,8)_ = 0.002, *p* = 0.968; treatment effect: *F*_(1,8)_ = 31.479, *p* = 0.001; treatment × sex interaction: *F*_(1,8)_ = 1.749, *p* = 0.223; male: *t*_(4)_ = 4.867, *p* = 0.008; female: *t*_(4)_ = 3.054, *p* = 0.038; **Figure 5B**), GAP-43 (sex effect: *F*_(1,8)_ = 3.593, *p* = 0.095; treatment effect: *F*_(1,8)_ = 37.893, *p* < 0.001; treatment × sex interaction: *F*_(1,8)_ = 1.578, *p* = 0.244; male: *t*_(4)_ = 3.171, *p* = 0.034; female: *t*_(4)_ = 5.837, *p* = 0.004; **Figure 5B**), and PSD-95 (sex effect: *F*_(1,8)_ = 1.074, *p* = 0.330; treatment effect: *F*_(1,8)_ = 36.889, *p* < 0.001; treatment × sex interaction: *F*_(1,8)_ = 1.154, *p* = 0.705; male: *t*_(4)_ = 5.016, *p* = 0.007; female: *t*_(4)_ = 3.716, *p* = 0.021; **Figure 5B**).

### Effect of MS on Sex Hormone Biosynthesis and Metabolism

**Figure 6:**
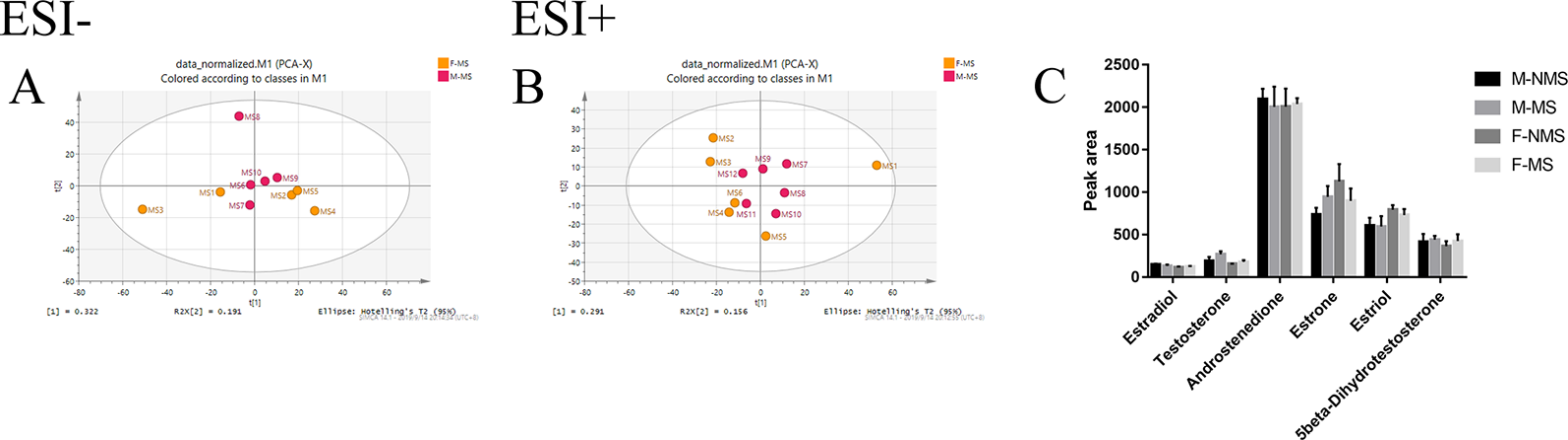
MS shows no gender difference in depression-like behavior in male and female rats. **(A)** PCA score plot of the F-MS and M-MS groups in negative ion mode. **(B)** PCA score plot of F-MS and M-MS groups in positive-ion mode. **(C)** Peak area of sex hormones and their metabolites detected by LC–MS/MS.

**Figure 6A** shows the negative ion mode PCA score plot of the F-MS and M-MS groups, and **Figure 6B** shows the positive-ion mode PCA diagram of the F-MS and M-MS groups. The results showed that F-MS and M-MS groups did not separate significantly. No statistical difference was detected in the peak area of sex hormones and their metabolites, including estradiol (male: *t*_(8)_ = 0.751, *p* = 0.474; female: *t*_(8)_ = 0.855, *p* = 0.417), testosterone (male: *t*_(8)_ = 1.344, *p* = 0.216; female: *t*_(8)_ = 1.016, *p* = 0.339), androstenedione (male: *t*_(10)_ = 0.338, *p* = 0.742; female: *t*_(10)_ = 0.141, *p* = 0.891), estrone (male: *t*_(10)_ = 1.410, *p* = 0.189; female: *t*_(10)_ = 0.920, *p* = 0.379), estriol (male: *t*_(10)_ = 0.070, *p* = 0.945; female: *t*_(10)_ = 0.702, *p* = 0.499), and 5beta-dihydrotestosterone (male: *t*_(10)_ = 0.256, *p* = 0.803; female: *t*_(10)_ = 0.605, *p* = 0.559) between the NMS and MS groups (**Figure 6C**). The specific information on these metabolites is listed in **Table 1**.

**Table 1:** Sex hormones and their metabolites.

NO	Scan Mode	Mass	RT	Metabolites	HMDB ID
M1	ESI-	272.1776	5.009	Estradiol	HMDB0000151
M2	ESI-	288.2089	10.78	Testosterone	HMDB0000234
M3	ESI+	286.1933	6.356	Androstenedione	HMDB0000053
M4	ESI+	270.162	6.363	Estrone	HMDB0000145
M5	ESI+	288.1725	5.797	Estriol	HMDB0000153
M6	ESI+	290.2246	10.272	5beta-Dihydrotestosterone	HMDB0006770

Determination of sex hormones and their metabolites of samples in negative ion and positive-ion modes via LC–MS/MS in HMDB database.

### Metabolomics on the molecular mechanisms of MS affecting depression-like behavior

**Figure 7:**
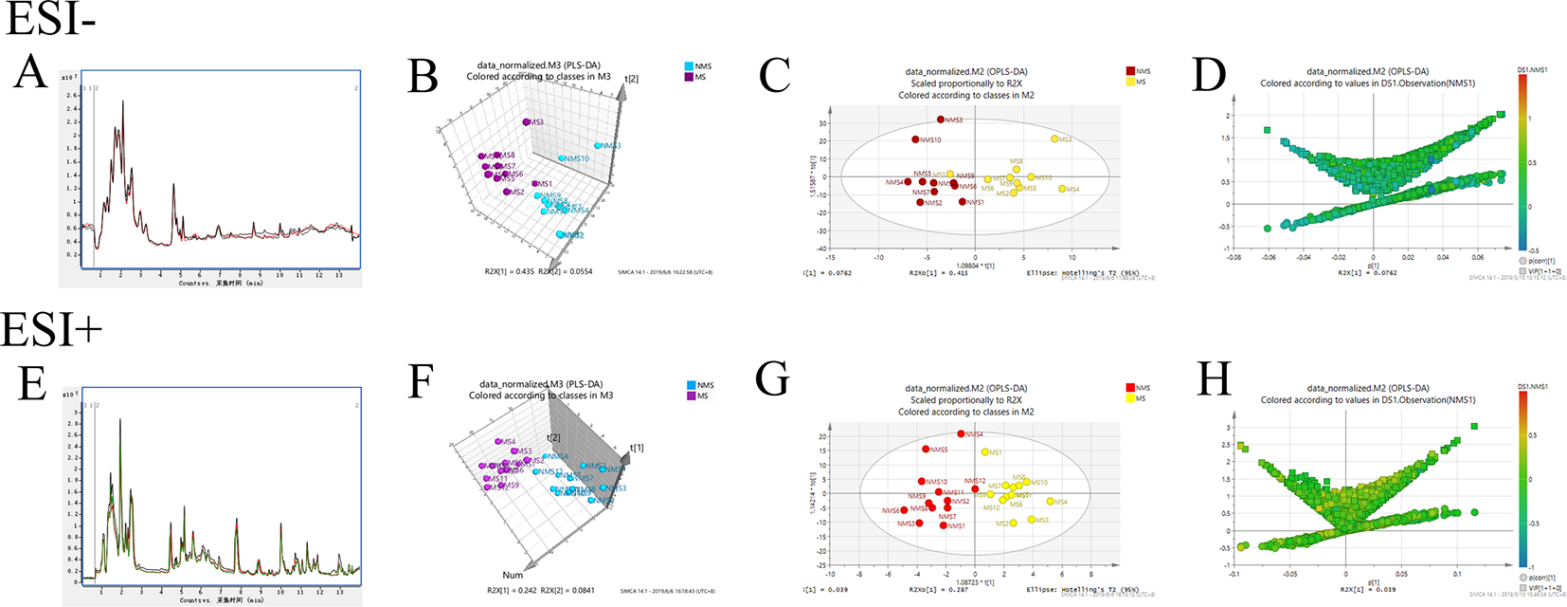
Model analysis of nontargeted metabolomics of brain tissues in SD rats. **(A)** TIC mass spectrum in negative ion mode. **(B)** PLS-DA 3D score plot in negative ion mode of the NMS and MS groups. **(C)** OPLS-DA score plot in negative ion mode of the NMS and MS groups. **(D)** The corresponding OPLS (V+S) plot of the NMS and MS groups in negative ion mode. **(E)** TIC mass spectrum in positive-ion mode. **(F)** PLS-DA 3D score plot in positive-ion mode of the NMS and MS groups. **(G)** OPLS-DA score plot in positive-ion mode of the NMS and MS groups. **(H)** The corresponding OPLS (V+S) plot of the NMS and MS groups in positive-ion mode.

**Figure 8:**
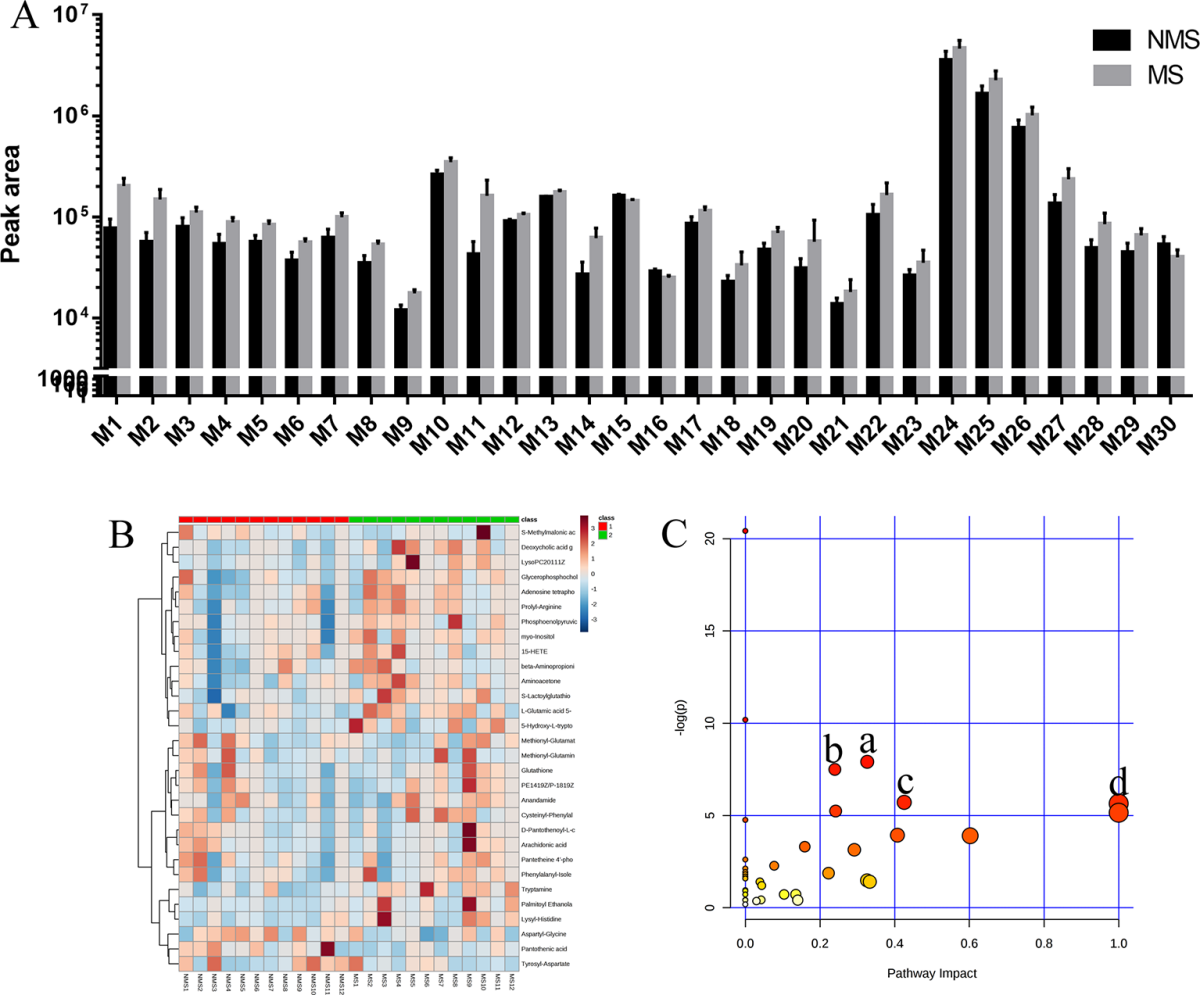
Analysis of differential metabolites. **(A)** Peak area detected by LC–MS/MS for differential metabolites in the brain tissue. **(B)** The relative content of differential metabolites from the heat map of brain tissues. Class 1: NMS group. Class 2: MS group. The ribbon −3~3: represents the content of differential metabolites from low to high. **(C)** Pathway analysis of differential metabolites in the NMS and MS groups. a: pantothenate and CoA biosynthesis, b: arginine and proline metabolism, c: glutathione metabolism, d: phenylalanine, tyrosine, and tryptophan biosynthesis.

Brain tissues were collected from the NMS and MS groups. Total ion current mass spectra were obtained at negative (**Figure 7A**) and positive-ion modes (**Figure 7E**) by LC–MS/MS metabolomics profiling. PLS-DA (**Figures 7B, F**) OPLS-DA (**Figures 7C, G**) and the corresponding OPLS (V+S) plots of NMS and MS groups (**Figures 7D, H**) were conducted to identify the differential metabolites and metabolic changes. The NMS and MS groups were evidently separated. The metabolite with features of VIP > 1 and *p* < 0.05 were considered potential significant differential metabolites. Subsequently, 30 endogenous metabolites were confirmed by comparing their mass spectra and chromatographic retention times with the available references, including MassBank, PubChem, and Human Metabolome Database. Their specific information is shown in **Table 2**. The peak area of the characteristic peaks for targeted metabolites is shown in **Figure 8A**. The biological functions of these differential metabolites were analyzed on MetaboAnalyst 4.0. **Figures 8B, C** show the results of heatmap and pathway analysis. Pantothenate and CoA biosynthesis (FDR = 0.0099; Impact = 0.33); arginine and proline metabolism (FDR = 0.0112; Impact = 0.24); glutathione metabolism (FDR = 0.0475; Impact = 0.43); and phenylalanine, tyrosine, and tryptophan biosynthesis (FDR = 0.0475; Impact = 1.00) were significantly altered metabolic pathways.

**Table 2:** Differential metabolites.

NO	Scan mode	Mass	RT	Metabolites	HMDB ID	MS/NMS
M1	ESI-	448.139	11.732	Deoxycholic acid glycine conjugate	HMDB0000631	↑
M2	ESI-	548.0627	11.757	LysoPC(20:1(11Z))	HMDB0010391	↑
M3	ESI-	72.0218	1.336	Aminoacetone	HMDB0002134	↑
M4	ESI-	586.1207	2.453	Adenosine tetraphosphate	HMDB0001364	↑
M5	ESI-	167.0246	2.088	Phosphoenolpyruvic acid	HMDB0000263	↑
M6	ESI-	270.058	2.954	Prolyl-Arginine	HMDB0029011	↑
M7	ESI-	255.988	1.889	Glycerophosphocholine	HMDB0000086	↑
M8	ESI-	179.0245	2.273	myo-Inositol	HMDB0000211	↑
M9	ESI-	378.0898	1.442	S-Lactoylglutathione	HMDB0001066	↑
M10	ESI+	161.1073	1.386	Tryptamine	HMDB0000303	↑
M11	ESI+	300.2829	7.726	Palmitoyl Ethanolamide	HMDB0002100	↑
M12	ESI+	228.1507	1.919	L-Glutamic acid 5-phosphate	HMDB0001228	↑
M13	ESI+	221.0253	1.248	5-Hydroxy-L-tryptophan	HMDB0000472	↑
M14	ESI+	284.2866	7.722	Lysyl-Histidine	HMDB0028953	↑
M15	ESI+	191.1338	4.906	Aspartyl-Glycine	HMDB0028753	↓
M16	ESI+	220.0002	1.86	Pantothenic acid	HMDB0000210	↓
M17	ESI-	69.0568	1.501	beta-Aminopropionitrile	HMDB0004101	↑
M18	ESI-	322.1007	5.983	D-Pantothenoyl-L-cysteine	HMDB0006834	↑
M19	ESI-	319.0512	1.977	15-HETE	HMDB0003876	↑
M20	ESI-	100.0161	5.865	(S)-Methylmalonic acid semialdehyde	HMDB0002217	↑
M21	ESI-	304.1441	6.355	Arachidonic acid	HMDB0001043	↑
M22	ESI+	278.1341	5.277	Methionyl-Glutamate	HMDB0028972	↑
M23	ESI+	278.0973	2.209	Methionyl-Glutamine	HMDB0028971	↑
M24	ESI-	363.0573	2.271	Anandamide	HMDB0004080	↑
M25	ESI-	307.0836	2.139	Glutathione	HMDB0000125	↑
M26	ESI-	358.0957	5.104	Pantetheine 4'-phosphate	HMDB0001416	↑
M27	ESI-	267.0954	4.725	Cysteinyl-Phenylalanine	HMDB0028782	↑
M28	ESI-	670.1369	1.564	PE(14:1(9Z)/P-18:1(9Z))	HMDB0008886	↑
M29	ESI-	278.0753	5.226	Phenylalanyl-Isoleucine	HMDB0028998	↑
M30	ESI+	296.1513	5.185	Tyrosyl-Aspartate	HMDB0029101	↓

Differential metabolites are confirmed in the NMS and MS groups based on VIP > 1. P < 0.05, ↑ shows upregulated metabolite. ↓ shows downregulated metabolite.

## Discussion

We used an MS model to investigate the effects of depression in adult SD rats with early adverse stress. The rats were weighed from PND28 to PND63. We observed that the weight gain of the MS group was significantly lower than that of the NMS group by analyzing the weight gain in this cycle, indicating that the MS model caused weight loss in SD rats. For specific data on food intake in rats, see **Supplement Material 1**. Ítalo Leite Figueiredo, DVM, PhD, believed that prolonged MS induced malnutrition status in rats (Figueiredo et al., 2016). The behavioral test results showed that sucrose preference (%) significantly decreased compared with the NMS group, indicating that MS contributed to anhedonia in SD rats. Sucrose and water consumption and percentage of sucrose and water consumption see **Supplementary Material 2**. The immobility time in forced swimming test significantly increased compared with the NMS group, revealing that MS contributed to the behavioral despair of SD rats. These findings indicated that the MS model caused depression-like behavior in SD rats. Central region time and distance in open-field test significantly decreased compared with the NMS group, whereas no significant statistical difference was found in the activity of the female group (**Figure 2E**). These data suggest that the MS model contributed to anxiety-like behavior in SD rats. All of these behavior results were in agreement with published results (Almeida-Suhett et al., 2017; Citraro et al., 2017). No statistical difference was found in gender between female and male rats. Clinical and experimental studies have shown that women are more likely to suffer from depression than men under exposure to early-life adversity (Duman et al., 2016; Mahmoud et al., 2016). ELS models in rodents cannot replicate the effects of gender factors. This condition may be due to the insensitivity of rodents to the means of behavior measurement and that female rodents are susceptible to differences in estrus cycles (Leussis et al., 2012; Goodwill et al., 2019). In this study, the cycle of MS was from PND 1 to PND 21, which was performed daily for 3 h in the morning and afternoon. The behavioral experiment started from PND 56. PND 56 to PND 63 denoted the adulthood of SD rats, and the estrogen levels of female rats in this period were relatively stable. Gender factors may not be an important factor for depression in the rodent ELS model. However, women with depression have a two times higher incidence than men. Future research and discussion on influence of gender factor on depression are required.

For synaptic plasticity, we observed the morphology and number of neurons in the hippocampus and prefrontal cortex through Nissl staining. The experimental results showed that the number of neurons in the CA1, CA2, and CA3 regions of the hippocampus and prefrontal cortex in the MS group was significantly reduced. The hippocampal DG region in the F-MS group was significantly reduced, and the M-MS group was insignificant compared with the NMS group. The MS model damaged the neurons in the hippocampus and prefrontal cortex and weakened the function of neuronal synthetic proteins. This result was consistent with the results of some studies. For depression, the depression group was associated with abnormal neuronal morphology, decreased hippocampal volume, and reduced pyramidal cells and granulocytes (Zhao et al., 2018). Changes in the size and density of neurons in the cortex and a decrease in the number of glial cells contribute to the development of depression (Bakhtiarzadeh et al., 2018). The effects of the MS model on the expression of synaptic-plasticity protein in the hippocampus and prefrontal cortex of adult SD rats were explored by immunohistochemistry. The experimental results showed that the MS group had lighter staining compared with the NMS group, and the percentage of positive area in the hippocampal CA1, CA2, CA3, DG, and prefrontal cortex was significantly reduced. This finding suggested that the MS model may cause adult-derived depression-like behavior in SD rats by regulating the expression of synaptic-plasticity proteins in the hippocampus and cortex. We evaluated the expression levels of the synaptic-plasticity protein markers PSD-95, SYN, and GAP-43 in the hippocampus and cortex by WB analysis. The experimental results showed that the expression levels of the three synaptic-plasticity proteins in the hippocampus and cortex of the MS group were significantly lower than that of the NMS group. This finding indicated that the MS model regulates synaptic plasticity and causes SD rats to suffer depression during adulthood. The same results showed that learned helplessness paradigm, as an accepted experimental model of depression, decreased the immunostaining of SYN, PSD-95, and GAP-43 in the CA3 region of the hippocampus of model animals. The results were opposite for animals treated with fluoxetine, revealing that the treatment of fluoxetine can modify the synaptic and axonal remodeling of the hippocampal CA3 region by learning helplessness models (Reines et al., 2008).

We used nontargeted metabolomics to detect metabolites in the brain tissue of SD rats to elucidate the underlying molecular mechanism of depression in the MS model by regulating synaptic plasticity. The results indicated that differential metabolites, including glutamine, aspartate, arginine, proline, L-glutamic acid 5-phosphate, glutamate regulate arginine, and proline metabolism, are considered to be associated with depression based on the pathway analysis of nontargeted metabolomics of brain tissue (Liu et al., 2019). Patients with depression show lower arginine levels compared with healthy controls (Moaddel et al., 2018). The NO levels in depression group was significantly lower than those in the normal group (Chrapko et al., 2004). Arginine is hydrolyzed to ornithine and urea by arginase in the urea cycle and oxidized to citrulline. Arginine is also converted by nitric oxide synthases to nitric oxide and citrulline in the nitric oxide cycle. NO acts as a messenger in physiological processes associated with depressive disorders (Wegener and Volke, 2010), especially on synaptic plasticity (Forstermann and Sessa, 2012). Asymmetric dimethylarginine, a potential endogenous factor that affects L-arginine levels, competitively inhibits eNOS and prevents NO production, which is related to depression and high arginine levels (Cooke and Ghebremariam, 2011; Hess et al., 2017). This result is consistent with our experimental results. L-proline is similar to GABA in terms of chemical structure and is a GABA mimetic. The accumulation of proline in GABAergic neurons can competitively inhibit glutamate decarboxylase, leading to the decrease in GABA production and affecting synaptic plasticity (Crabtree et al., 2016). The levels of proline in the MS group significantly increased in our results. Three differential metabolites, namely, pantothenic acid, D-pantothenoyl-L-cysteine, and pantetheine 4'-phosphate, enrich the pathway for pantothenate and CoA biosynthesis. Decreased pantothenic acid levels and increased pantetheine 4'-phosphate levels in the MS group may affect CoA synthesis. CoA plays an essential role in the metabolism of carboxylic and fatty acids (Leonardi et al., 2005). High CoA levels in the mitochondria can increase ATP synthesis, increasing the glutathione levels, inhibiting inflammation, and reducing oxidative stress to promote depression (Nitto and Onodera, 2013; Slyshenkov et al., 2004; Wojtczak and Slyshenkov, 2003). The glutathione metabolism pathway is involved in glutamate, gamma-glutamylcysteine, glutathione, cysteine, glycine, and gysteinylglycine. Glutathione is a potential marker of depression in early stage (Freed et al., 2017). Impaired synaptic plasticity is associated with low levels of glutathione (Almaguer-Melian et al., 2000). Phenylalanine is increased and tyrosine is decreased in the enrichment pathway of phenylalanine, tyrosine, and tryptophan biosynthesis. High phenylalanine concentration and phenylalanine/tyrosine ratio are associated with neopterin concentrations in patients suffering from inflammation (Vancassel et al., 2018; Korte-Bouws et al., 2019), which can be mediated by synaptic-plasticity regulation.

In this study, no significant separation was found between the male and female groups in the MS group by PCA analysis. No significant statistical difference was found in the biosynthesis and metabolism of sex hormones between NMS and MS group based on the LC–MS/MS analysis of the peak area of estradiol, testosterone, androstenedione, estrone, estriol, and 5β-dihydrotestosterone. This condition indicated that the MS caused depression-like behavior in adult male and female rats were similar. A rational rodent model should be developed to investigate the effects of gender factors in depression research.

## Conclusion

The experimental results showed that the MS model of SD rats can lead to depression-like behavior in adulthood. The molecular mechanism to regulate synaptic plasticity may be related to arginine and proline metabolism; pantothenate and CoA biosynthesis; glutathione metabolism; and phenylalanine, tyrosine, and tryptophan biosynthesis. However, gender did not interfere with depression-like behaviors in adult MS rats.

## Data Availability Statement

The datasets analyzed in this article are not publicly available. Requests to access the datasets should be directed to YC, 1293332045@qq.com.

## Ethics Statement

The animal study was reviewed and approved by Committee of Animal Experiment Ethics Review in Guangzhou University of Chinese Medicine.

## Author Contributions

RZ and YS: designed the study. YC, HL, SC, CS, WW, HM, ZD, SB, and LY: performed the experiments. KC: analyzed the data. YC: wrote the manuscript.

## Funding

This work was supported by the National Natural Science Foundation of China (No. 81873271. 81573912), Key project of Educational Commission of Guangdong Province of China (NO.2018KZDXM020), and Scientific Research Team Training Project of GZUCM (NO.2019KYTD301).

## Conflict of Interest

The authors declare that the research was conducted in the absence of any commercial or financial relationships that can be construed as a potential conflict of interest.

## Supplementary Material

Table S1 Food intake for each group from PND28 to PND62 (g, n = 15).

Figure S1 Daily food intake on average for each rat from PND28 to PND62.

Figure S2 Consumption of sucrose and water in the sucrose preference test.

Figure S3 Percentage of consumption sucrose and water in the sucrose preference test.
