# Supplementary material for: Early-Life Stress Induces Depression-Like Behavior and Synaptic-Plasticity Changes in a Maternal Separation Rat Model: Gender Difference and Metabolomics Study
Source: Front Pharmacol. 2020 Feb 26;11:102. doi: 10.3389/fphar.2020.00102 (PMC7055479; doi:10.3389/fphar.2020.00102)
Supplement: Table S1 — Food intake for each group from PND28 to PND62 (g, n = 15). [file Table_1.docx]

**Table** **S1** **Food** **intake** **for** **each** **group** **from** **PND28** **to** **PND62** **(g,** ***n* = 15)**

Daily food intake for each group from PND28 to PND62 are displayed in the table above (*n* = 15).

| **Table S1 Food intake for each group from PND28 to PND62 (g, n=15)** | | | | |
| --- | --- | --- | --- | --- |
|  | M-NMS | M-MS | F-NMS | F-MS |
| PND28 | 209.1 | 203 | 167.5 | 168.8 |
| PND35 | 224.1 | 218.9 | 174.7 | 179.4 |
| PND42 | 230.9 | 235.1 | 169 | 168.5 |
| PND49 | 226 | 229 | 169.2 | 175.7 |
| PND56 | 239.2 | 242.3 | 174.6 | 167.3 |
| PND62 | 240.1 | 233.8 | 169.6 | 174.6 |
