# Supplementary figures and images for: Early-Life Stress Induces Depression-Like Behavior and Synaptic-Plasticity Changes in a Maternal Separation Rat Model: Gender Difference and Metabolomics Study
Source: Front Pharmacol. 2020 Feb 26;11:102. doi: 10.3389/fphar.2020.00102 (PMC7055479; doi:10.3389/fphar.2020.00102)

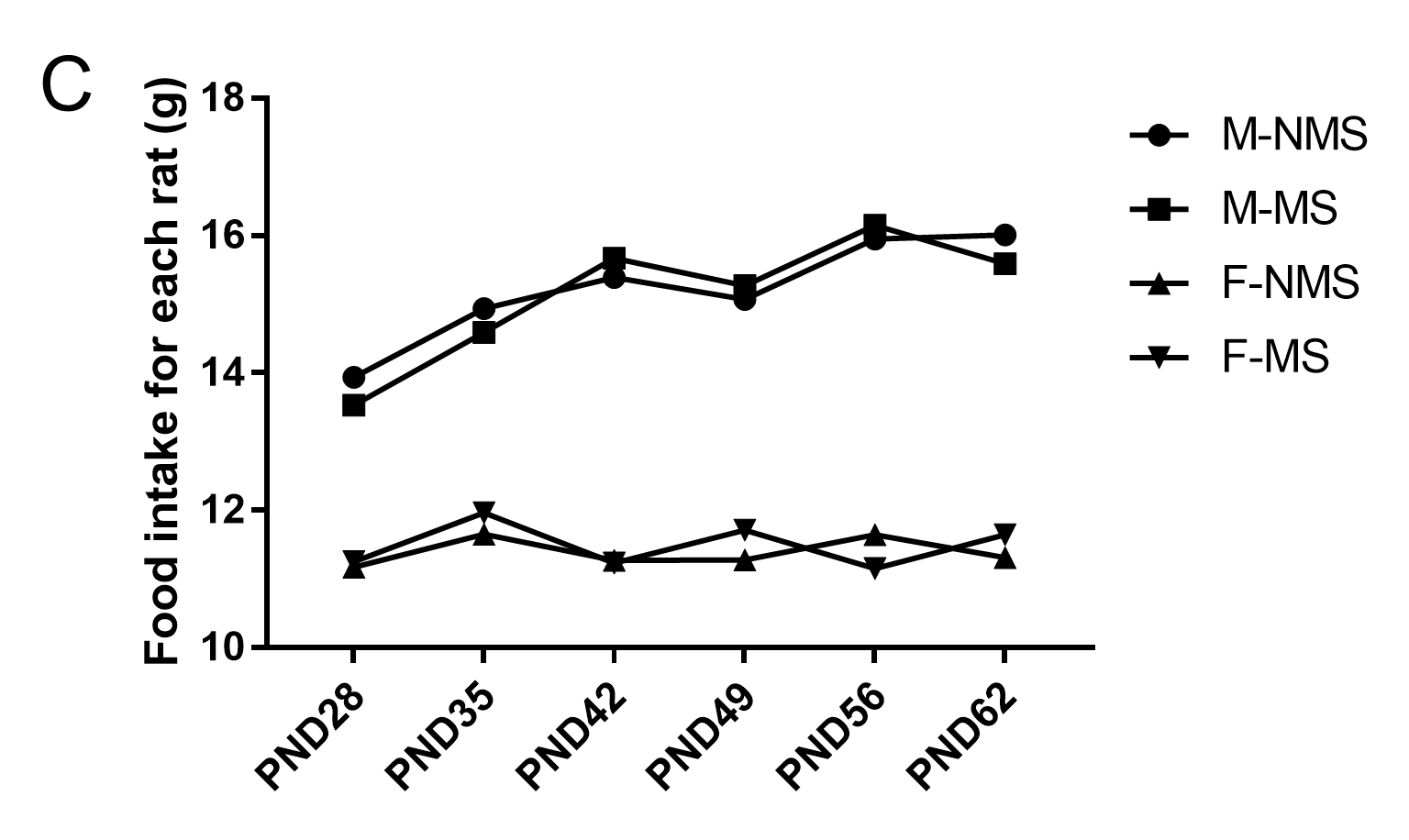

Supplement: Figure S1 — Daily food intake on average for each rat from PND28 to PND62. [file Image_1.tif]

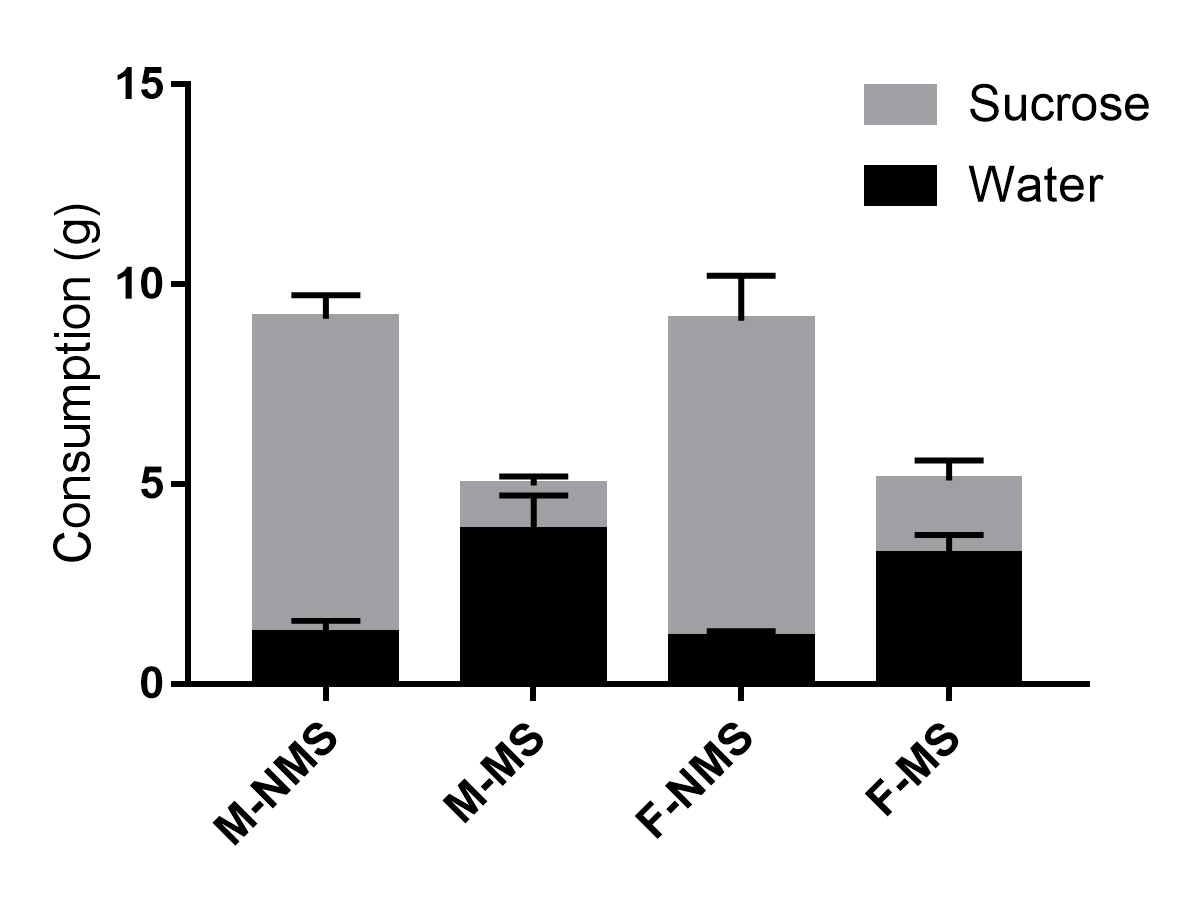

Supplement: Figure S2 — Consumption of sucrose and water in the sucrose preference test. [file Image_2.tif]

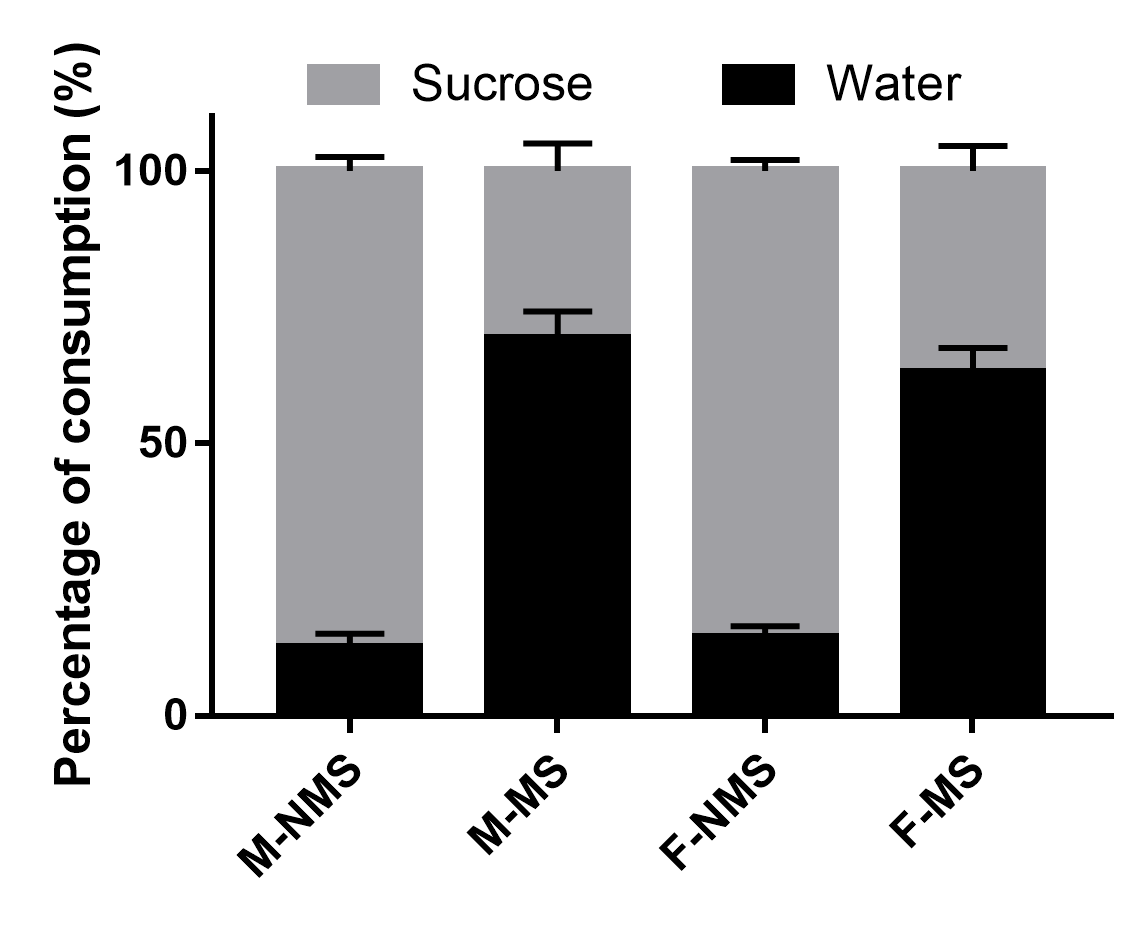

Supplement: Figure S3 — Percentage of consumption sucrose and water in the sucrose preference test. [file Image_3.tif]
